# Elective course “Climate-sensitive health counselling” – prevention as an opportunity for people and planet? An interactive, student-led project focusing on prevention and agency in physician’s climate communication

**DOI:** 10.3205/zma001616

**Published:** 2023-05-15

**Authors:** Hannah Fülbert, Louis N. Schäfer, Laura M. Gerspacher, Stefan Bösner, Christina Schut, Ralph Krolewski, Michael Knipper

**Affiliations:** 1 Justus Liebig University Giessen, Medical Faculty, Giessen, Germany; 2 Health for Future Giessen, Giessen, Germany; 3 Philipps University Marburg, Medical Faculty, Marburg, Germany; 4 Health for Future Marburg, Marburg, Germany; 5 Philipps University Marburg, Department of General Practice/Family Medicine, Marburg, Germany; 6 Justus Liebig University Giessen, Institute of Medical Psychology, Giessen, Germany; 7 Academic teaching practice of the University of Cologne, Gummersbach, Germany; 8 Justus Liebig University Giessen, Institute for History of Medicine, Giessen, Germany

**Keywords:** climate change, global warming, carbon footprint, conservation of natural resources, sustainable development, global health, primary prevention, health communication, undergraduate medical education, planetary health

## Abstract

**Objective:**

According to the WHO, anthropogenic climate change poses the greatest threat to human health in the 21^st^ century. However, the link between climate change and human health is not an integral part of medical education in Germany. Within a student-led project, an elective clinical course was designed and successfully implemented, which has been made accessible to undergraduate medical students at the Universities of Giessen and Marburg. The implementation and didactic concept are explained in this article.

**Methodology:**

In a participatory format, knowledge is imparted using an action-based, transformative approach. Topics discussed are, amongst others, interactions of climate change and health, transformative action, and health behavior, as well as “green hospital” and the simulation of a “climate-sensitive health counselling”. Lecturers from different disciplines within and beyond medicine are invited as speakers.

**Results:**

Overall, the elective was evaluated positively by the participants. The fact that there is a high demand among students for participation in the elective, as well as for the transfer of concepts underlines the need for including this topic into medical education. The implementation and further development of the concept at two universities with different study regulations demonstrates its adaptability.

**Conclusion:**

Medical education can raise awareness of the multiple health consequences of the climate crisis, can have a sensitizing and transformative effect on various levels, and can promote climate-sensitive action ability in patient care. In the long term, however, these positive consequences can only be guaranteed by including mandatory education on climate change and health in medical curricula.

## Zusammenfassung

**Zielsetzung:**

Der anthropogene Klimawandel ist laut der WHO die größte Gefahr für die menschliche Gesundheit im 21. Jahrhundert. Dennoch stellen die Zusammenhänge zwischen Klimawandel und Gesundheit keinen festen Bestandteil der ärztlichen Ausbildung dar. Im Rahmen einer studentischen Initiative wurde ein klinisches Wahlfach konzipiert und durchgeführt, welches für Studierende der Universitäten Gießen und Marburg zugänglich ist. Durchführung und didaktisches Konzept sollen in diesem Beitrag erläutert werden.

**Methodik:**

In einem partizipativen Format werden Inhalte mit einem praktischen, transformativen Ansatz besprochen. Es werden zunächst die Zusammenhänge zwischen Klimakrise und Gesundheit aufgezeigt, dann u.a. das transformative Handeln und Gesundheitsverhalten behandelt, die anschließend im Bereich Green Hospital und der Simulation einer „Klimasprechstunde“ münden. Dazu werden Dozierende unterschiedlicher Fachrichtungen aus der Medizin sowie aus anderen Bereichen eingeladen.

**Ergebnisse:**

Das Wahlfach wird insgesamt positiv bewertet. Der Umstand, dass die Nachfrage sowohl zur Teilnahme am Wahlfach als auch zur Weitergabe von Konzepten hoch ist, unterstreicht den Bedarf an Lehre zur Thematik. Die Durchführung und Weiterentwicklung des Konzepts an zwei Universitäten mit verschiedenen Studienordnungen zeigt dessen Adaptivität auf.

**Schlussfolgerung:**

Medizinische Lehre kann Bewusstsein für die mit der Klimakrise zusammenhängende Gesundheitsrisiken schaffen, transformativ wirken, sowie einen präventiven Ansatz der Gesundheitsversorgung fördern. Langfristig können diese positiven Effekte allerdings nur durch eine Aufnahme des Themenkomplexes Klimawandel und Gesundheit in die Curricula gewährleistet werden. Das Projekt zeigt, dass entsprechende Angebote realisierbar sind und gut angenommen werden.

## 1. Introduction

Anthropogenic climate change has been characterized as the greatest threat for human health in the 21^st^ century by the World Health Organization (WHO) [1]. Worldwide, our use of fossil resources is reaching natural boundaries [2] which has a vast impact on our health: droughts, heat waves, floods, rising sea levels, the increasing spread of disease vectors and infectious diseases, as well as psychological trauma are just a few examples of health risks resulting from climate change [3]. At the same time, the German healthcare sector itself causes about 5% of all national greenhouse gas emissions [4] and will thus not only have to deal with the consequences of climate change in the future but also actively exacerbates them. If the entire global healthcare system were a country, it would rank fifth among the largest global CO_2_ emitters [5]. Medical personnel enjoy great trust within the population, which is why this professional group holds a particularly important mediating role regarding climate change mitigation [6], [7]. By providing information and advice to their patients, future physicians can make an important contribution to the prevention of the negative health effects of climate change and to raising awareness about the issue [8], [9]. In addition, the healthcare professions play a key role in decarbonizing the healthcare system regarding structures and processes in doctor’s offices and hospitals [10], [11]. Yet, this can only succeed if the topics of climate change and planetary health are made mandatory in the education of future health professionals [12], [13]. However, most teaching on the topic is only optional, and the emphasis in medical education still tends to be on disease treatment rather than prevention and a consistent inclusion of psychosocial and environmental contextual factors [14]. The results of a recent study confirm this gap: According to El Omrani et al. only 15% of medical schools worldwide currently offer teaching on climate change [15]. 

The present project addresses precisely this issue: Through sound knowledge transfer, practical exercises and continuous reflection, medical students are to be prepared for their important mediating role as future physicians and sensitized for the topic. At the Universities of Giessen and Marburg, the elective “Climate-sensitive health counselling” is an important first step towards integrating the topic of climate change and health into the medical curriculum and imparting relevant skills related to the topic. For now, the teaching project is still a student-led elective course, but in the long term, the aim is a compulsory format for all medical students. With the following project description, we aim to describe the implementation of this elective course and to share experiences that can serve as guidance for the development of similar projects.

## 2. Project description

### 2.1. Development of the project

The idea for the elective course “Climate-sensitive health counselling” emerged in June 2020 following an online lecture series on “planetary health” by the German Alliance on Climate Change and Health (KLUG e.V.), which was attended by students of the Justus Liebig University (JLU) Giessen as part of the special track curriculum (Schwerpunktcurriculum, SPC) Global Health [16]. In this online course, a general practitioner presented his concept of a climate-sensitive consultation [17]. The already noticeable consequences of the climate crisis and its close connection to the health sector, as well as the voluntary work of some students at Health for Future (H4F) motivated the founding group of the elective – initially consisting of six medical students from the JLU Giessen and the Philipps University Marburg – to develop an educational offer for students that teaches the theoretical and practical basics of the topic (see attachment 1 ).

#### 2.2. Learning objectives

**Figure 1:**
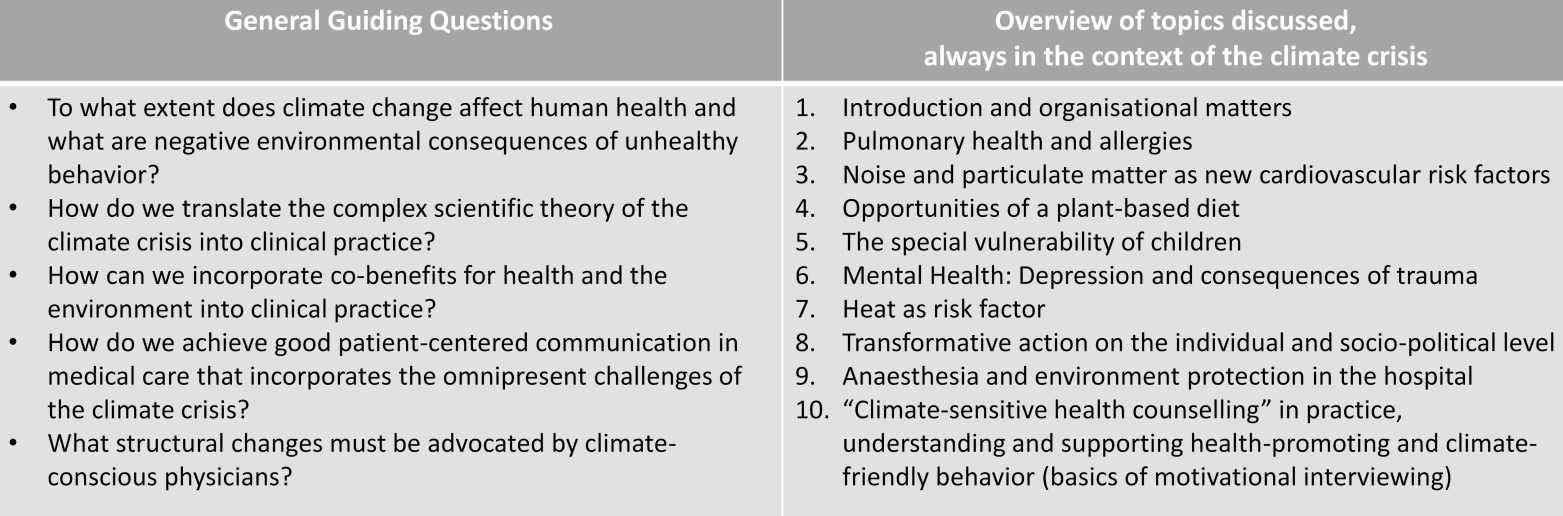
Exemplary overview of the general guiding questions underlying the elective as well as the topics discussed. The choice of topics differs slightly between the individual semesters.

The aim of the elective course is to make the health sector more sustainable and equip future health professionals with the skills and knowledge needed to conduct interactive patient consultations in the context of the climate crisis. The course also takes into account individual socio-economic factors, as illustrated in figure 1 (Fig. 1) and detailed in the concept and schedule provided in attachment 2 and attachment 3 for the winter semester of 2021/22. After having completed the elective, students should understand the fundamentals of climate change, important anthropogenic drivers, as well as resulting health consequences across medical specialties. A particular emphasis is placed on knowledge of behaviors that are detrimental to health and climate [18]. This awareness should then help in later medical consultations to reconsider habits which harm both climate and health together with patients, to advise them in a motivating and cooperative manner, and to improve health and quality of living in the long-term. In addition, the course is intended to provide a sociopolitical perspective aiming to motivate students and show them ways to advocate for the issue beyond medicine to initiate a necessary overall societal transformation towards more climate protection. For a green transformation in hospitals, the elective imparts knowledge about emission sources in healthcare and concrete problem-solving approaches. The selection of topics is based on the learning objectives on planetary health anchored in the updated National Competence-Oriented Catalogue of Learning Objectives in Medicine (Nationaler Kompetenzbasierter Lernzielkatalog Medizin NKLM) [https://www.nklm.de] which is to be introduced at all German universities in the coming years.

#### 2.3. Scope and structure

The elective course “Climate-sensitive health counselling” started in the winter term 2020/21 at the JLU Giessen and is also open to all medical students at the University of Marburg, a neighboring town, as a clinical elective since the summer term 2021. The target group are undergraduate medical students in the clinical part of their studies (5^th^ semester and above). The maximum number of participants is 25. The elective is organized by a team of students who are supported by two professors. In Giessen, the course is offered once a week during the semester and comprises a total of nine seminar sessions of 90 minutes each, as well as one concluding session of six hours. In accordance with the study regulations of the University of Marburg, the elective course has been extended to three semester hours per week there (see attachment 4 ), adding thematically relevant videos from the Planetary Health Academy [https://planetary-health-academy.de/] of KLUG e.V. [19]. The selection of videos includes for example “transdisciplinary perspectives”, “gender and global south perspective”, “social tipping elements” as well as climate communication. In addition, two sessions for reflection, discussion and clarification of open questions take place in Marburg.

**Figure 2:**
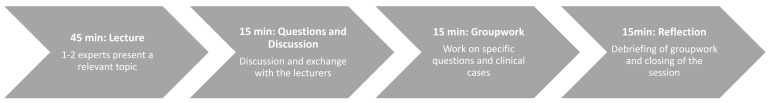
General organization of the sessions (except concluding session).

The seminars are always structured in a similar way: Two students of the organizational team prepare and moderate the session and document what has been discussed. Usually, each seminar starts with a short 45-minute presentation about a relevant topic presented by one or two invited experts (see figure 2 (Fig. 2)). The focus is always on the scientific soundness of the content, prevention work and appropriate communication with patients. Subsequently, the topic is discussed in the group. The course concludes with groupwork in smaller groups of three to four students. To guide the discussions and promote interaction and reflection, the organizational team prepares specific guiding questions for each session, as well as case studies with climate relevance from everyday clinical practice.

#### 2.4. Didactic concept

##### 2.4.1. Interactive format and teaching at eye level

The didactic approach of the elective is characterized by participation implemented through discussion, group work and teaching at eye level. This is mainly achieved by avoiding teacher-centered teaching and giving enough time for exchange between the students and the lecturer after each theoretical input (see figure 2 (Fig. 2)).

##### 2.4.2. From theory to practice

To sustainably consolidate the course material and to promote individual learning success in the long term, the elective combines theoretical knowledge, discussions, clinical case studies and practical units on successful interviewing. The latter is taught particularly during the final six-hour session: After the presentation of relevant aspects of different health behavior models and basic knowledge on motivational interviewing by a psychologist working in the field of medical psychology, practical insights into climate-sensitive health counselling are provided by a general practitioner. Subsequently, climate-sensitive dialogues with patients, applying a medical case vignette, are simulated and reflected on in small groups. The students can put the various aspects of the elective course into practice by trying them out directly in conversations. Furthermore, the students carry out a self-experiment throughout the semester during which they replace one everyday habit with a more climate-friendly alternative. At the end of the elective, the experience is reflected in small groups, as well as in the class. In future patient counselling this experience can become valuable in terms of a change in perspective.

##### 2.4.3. An interdisciplinary and inter-professional approach

The elective is designed to be interdisciplinary and interprofessional. The discussion of topics in medicine spans across various specialties with a particular focus on environmental factors, with the goal of enhancing and broadening the students’ pre-existing knowledge. The cooperation with the Institute of Medical Psychology of JLU Giessen and lecturers from scientific institutions such as the German Meteorological Service (Deutscher Wetterdienst) or the Helmholtz Zentrum München provide a holistic view of the topic beyond medicine. For the sake of making the healthcare system more climate-friendly, transformative action and avoidable emissions within the healthcare system are also addressed.

##### 2.4.4. Proof of performance

Adapted to the specific institutional requirements, the proofs of performance differ between students from Marburg and Giessen. In Marburg, a three to five-page essay represents the proof of performance. For that purpose, the students either choose the topic themselves or can refer to a list of suggested topics provided by the organizing team. The topic is then to be discussed based on an individually posed research question. Students from Giessen, on the other hand, summarize one seminar unit with a focus on relevant aspects for later patient consultations. Furthermore, two current publications on the topic are to be researched. These papers are later summarized in one script and made available to all participants.

#### 2.5. Long-term establishment, networking, and cooperation

The selection of local speakers is given special attention. On the one hand, to establish permanent teaching on the most important health crisis of the 21^st^ century, on the other hand, to build long-term collaborations. In Giessen, this is an important contribution to the overall university sustainability strategy. In addition, the elective course is closely related to other events on climate change and planetary health, such as the “Model-World Health Assembly” on “Global Agenda on Health, Environment and Cimate Change”, organized by the WHO (Geneva), the American University Beirut and the SPC Global Health Giessen in 2021 and 2023. In Marburg, the lecture series "Climate Crisis and Health" was developed in cooperation between H4F Marburg, the Dean's Office of the Faculty of Medicine and the Green Office initiative, based on the elective. In addition, there is a close connection to KLUG e.V. and the H4F local groups. Both can be important starting points for socio-political commitment and enable students to get involved in climate protection beyond the academic context, for example at their workplace or in local politics. 

#### 2.6. Evaluation

The elective is continuously evaluated. In the course of each semester, two surveys are made available via the virtual learning platform k-med (see attachment 5 ), enabling participants to rate each individual session according to various aspects on a scale from “very poor” (1) to “very good” (5), as well as to provide free-text feedback. In addition, the continuous dialog between the team and participants provides the opportunity to make verbal suggestions for improvement at any time. The primary goal is to create space for reflection and constructive criticism to identify potential for improvement and to further develop this newly established course. 

## 3. Results

### Evaluation: Feedback from the participating students

The fact that all 25 available places (15 for Giessen and 10 for Marburg students) were booked in the first semesters serves as confirmation of the substantial need and interest for teaching on the subject of climate change and health in Germany. Table 1 (Tab. 1) gives an overview of the evaluations of the individual sessions from the three semesters winter 2020/21, summer 2021 and winter 2021/22. On average, all sessions were rated at least as “good” (4), but generally between “good” (4) and “excellent” (5) (see table 1 (Tab. 1)). Based on the overall evaluation, the high level of motivation reported by participants to continue dealing with the subject matter should be emphasized. The relatively low average rating for the increase in knowledge can possibly be explained by the fact that many participants already dealt with the topic before attending the elective and often already had extensive pre-existing knowledge. Overall, a selection bias can be assumed in the evaluation, since participation in the evaluation is voluntary.

Based on the evaluation, sessions with potential for improvement were identified and revised for the coming semesters. Furthermore, the selection of topics was extended together with the participants: Among others, “climate protection in hospitals” and “pediatrics and climate” were additionally included. The design of the concluding six-hour session was also adapted. In addition, lecturers were informed of the students' prior knowledge to avoid frequently criticized redundancies which occurred in the first two semesters, especially regarding the basics of climate change. Furthermore, time management in the sessions was optimized. 

## 4. Discussion

Even though the elective is well received by the participants, its long-term benefit cannot yet be assessed. This is in particular due to the fact that most of the former participants have still not entered the workforce and thus, the knowledge and interview techniques acquired in the elective are not applied in daily patient counselling yet. However, the feedback indicates that the participants are highly motivated to incorporate what they have learned into their future medical work and are eager to continue dealing with the topic. Yet, for objective and meaningful results, further studies and surveys are needed, for example on the extent to which former participants are committed to the topic in the long term and actually implement the acquired skills in their subsequent professional lives. Nevertheless, the fact that some general practitioners already offer climate-sensitive health counselling can be seen as a sign that the concept is suitable for practice and that sound training on the topic is highly relevant [20], [21]. To ensure a nationwide offer of climate-sensitive health counselling in the future, the presented project aims to foster the integration of the topic in general medical education for preparing undergraduate medical students in the best possible way. In addition, the elective is intended to encourage students to incorporate the topics of sustainability and climate protection into their own activities at various levels. This applies both to the individual level and to action in institutions and politics. It thus addresses the idea anchored in the guiding principle “think globally – act locally”, that local action and commitment can influence processes on a larger global level and aims at the associated individual capacity to act [22]. Thus, it addresses in a synergistic way the following United Nations Sustainable Development Goals (SDGs) for the global promotion of peace, dignity and equality at the economic, social and environmental levels: SDG 3 (good health and well-being), SDG 4 (quality education), and SDG 13 (climate action), SDG 14 (life below water) and SDG 15 (life on land) [23].

However, the integration of the topic into medical training can only have a long-term perspective if it is included as compulsory component in the catalogue of learning objectives and medical curricula. It should also be emphasized that continuity can only be ensured through institutionalization. This means that courses on the topic must be designed and organized by university staff and cannot depend solely on the voluntary commitment of some individuals, especially students. Yet, teaching offers such as the described elective or the mentioned Planetary Health Academy can serve as important starting points. Moreover, they can sensitize both teachers and faculty to the topic of planetary health and its integration into medical education. At the Faculty of Medicine in Giessen, for example, this initiative has triggered greater consideration of the topic in curricular development. In particular, the possibility of positively influencing both personal and planetary health through lifestyle changes in the sense of so-called “health co-benefits” can be seen as a great opportunity for human health. Whether it's “biking instead of driving” or favoring a predominantly plant-based diet: Such and other habit changes are healthy and reduce CO_2_ emissions [17], [24], [25]. Prevention as a climate protection measure must therefore become an integral part of medical training.

## 5. Conclusion

Education creates awareness, promotes preventive measures, and enables people to take action [26], [27]. The resulting self-efficacy expectation is currently needed more than ever to diminish the consequences of climate change and to find a path to a climate-friendly future [28]. The presented elective is a small step in this direction, towards greater awareness and knowledge transfer in the health sector. The feedback from the first three semesters was very positive and students expressed great interest in the subject. In addition, some former participants are now actively involved in the organization of the course and contribute their ideas there. Apart from raising awareness among future physicians, the elective successfully sensitized some lecturers for the topic. In preparation for their lecture, some of them dealt intensively with the links between their field and the climate crisis for the first time, which was evaluated as motivating and insightful. Not least due to the award of the “Hessian University Prize for Excellence in Teaching” 2022, the elective has also already been in the focus of local media [29], [30], [31].

While the elective course “Climate-sensitive health counselling” was one of the first of its kind in Germany in the winter of 2020, other initiatives have since emerged at German universities, and the students from Giessen and Marburg involved in the organization have become contact persons for teachers and students. The recently updated NKLM 2.0 which emphasizes the nexus climate change and health, gives reason to hope that the topic will finally find its way into the training and continuing education of physicians and that a change in thinking is currently being initiated on a larger scale [13]. The elective “Climate-sensitive health counselling” can thus be seen as a transitional project towards institutionalized and mandatory teaching formats. This is necessary to train competent and empathetic physicians and multipliers of climate-friendly change, and to protect human lives.

## Acknowledgements

Our special thanks go to the entire organizing team of the elective, which has developed and established the project and keeps it alive: Hanna Burow, Magdalene Denneler, Jonas Derben, Charlotte Friedrich, Miriam Hobbhahn, Annamaria Jaschke, Carina Körner, Hannes Kreissl, Leonard Maier, Magdalena Maurer, Anne Maushagen, Lisa Nieberle, Sibel Savas, Theresa Scheftschik, Emma Lou Tischbier and Antonia Weigel. We would further like to thank all the lecturers who supported the elective subject by volunteering to give lectures: Prof. Dr. Johannes Kruse, Dr. Stefan Kuhnert, Dr. Ferdinand Lehmann, Prof. Dr. Klaus-Peter Zimmer, Dr. Eleonore Heil (all JLU Gießen / UKGM), Dr. Alexandra Schneider (Helmholtz Zentrum München), Dr. Sebastian Göbel (Praxis grüne Aue, Hermaringen), Prof. Dr. Ursel Heudorf (Gesundheitsamt Frankfurt a.M.), Prof. Dr. Thomas Münzel (JGU Mainz), Prof. Dr. Christian Witt (Charité Berlin), Prof. Dr. Andreas Matzarakis (Deutscher Wetterdienst), Dr. Christoph Thesen (Hochschule Fulda), Dr. Sylvia Hartmann, Dr. Martin Herrmann, Friederike von Gierke (all KLUG e.V.), Matthis Keil (Universität Bremen), Dr. Alina Herrmann, Claudia Quitmann (both Heidelberg Institute of Global Health), Dr. Christian Grah (Klinikum Havelhöhe) as well as Prof. Dr. Claudia Traidl-Hoffmann (Universität Augsburg/Helmholtz Zentrum München), Prof. Dr. August Stich (Universität Würzburg), Dipl.-Psych. Katharina Mikus (Psychologists For Future), Dr. Antje Herbst, Dr. Kerstin Bäumer, and Dr. Werner Fleck. Finally, Hannah Otto representing the team of the Planetary Health Academy, the team of the dean’s office of the Faculty of Medicine of the PU Marburg for their intensive support in adapting the elective to the study regulations there, the Dean of Studies of the Faculty of Medicine of the JLU Giessen, Irene Serrano from the SPC Global Health at JLU Giessen, Dr. Elisabeth Szabo from the Department of General Practice/Family Medicine at the PU Marburg, as well as Joe Berns, Lena Mähnß and Philip Reich who helped translating this article.

## Author contributions

Hannah Fülbert, Louis N. Schäfer and Laura M. Gerspacher contributed equally to this work and share joint first authorship.

Stefan Bösner, Christina Schut, Ralph Krolewski and Michael Knipper critically reviewed the manuscript and provided helpful feedback on how to improve it. 

## Competing interests

The authors declare that they have no competing interests. 

## Supplementary Material

Initial concept paper of the elective “Climate-sensitive health counselling”, Giessen, for the winter term 2020/21 [translated from the original German version]

Concept and performance record of the elective “Climate-sensitive health counselling” Giessen; winter term 2021/22 [translated from the original German version]

Schedule of the elective “Climate-sensitive health counselling” Giessen, winter semester 2021/22 [translated from the original German version]

Expanded concept of the elective “Climate-sensitive health counselling”, Marburg, winter semester 2021/22 [translated from the original German version]

Exemplary survey instrument for the evaluation of the elective “Climate-sensitive health counselling”, summer semester 21

## Figures and Tables

**Table 1:**
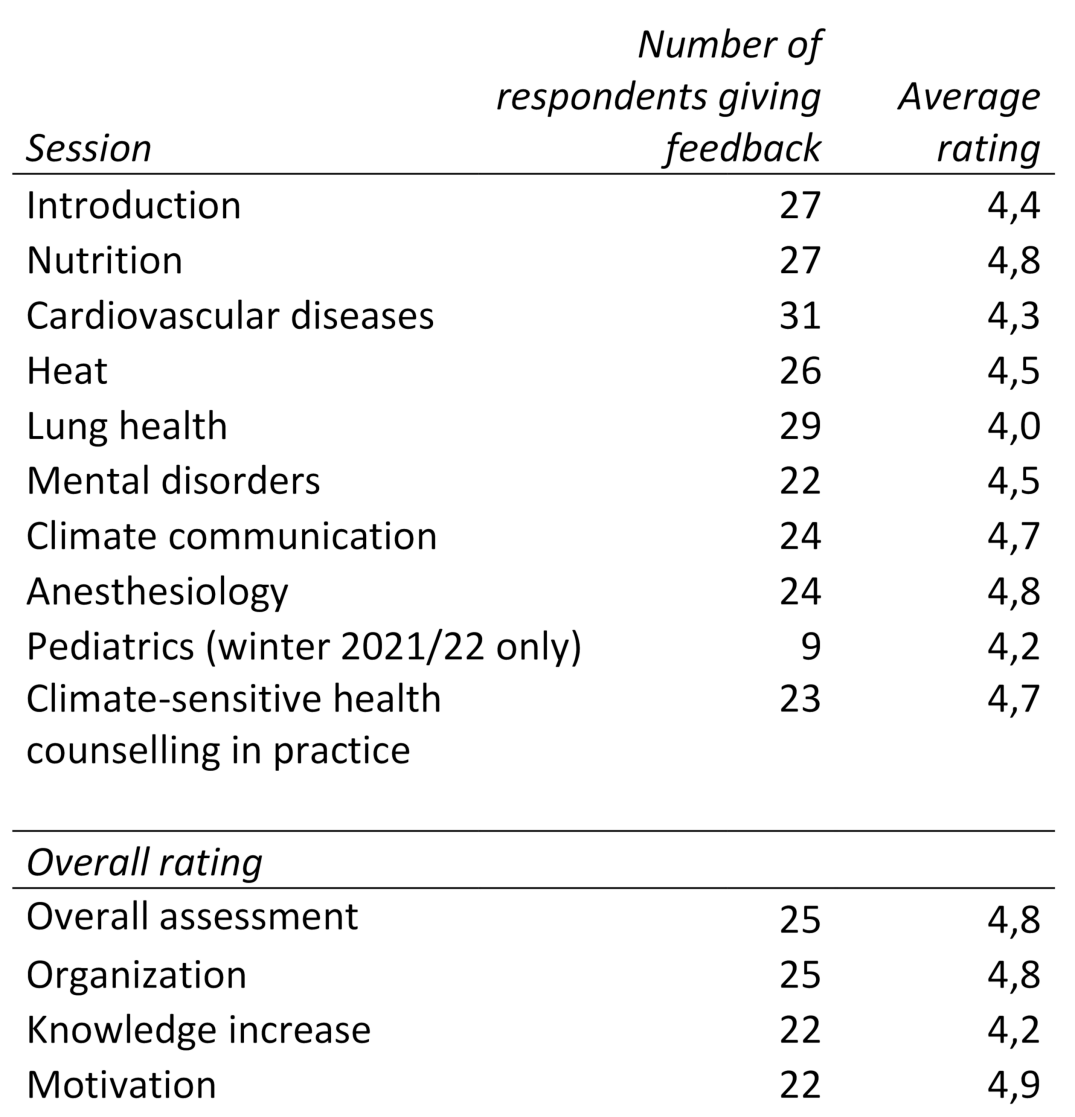
Average ratings of the sessions summed up from winter semester 2020/21, summer semester 2021 and winter semester 2021/22 as well as the number of those who participated in the evaluation. Rating scale: excellent (5), good (4), average (3), bad (2), terrible (1).
