# Supplementary material for: Elective course “Climate-sensitive health counselling” – prevention as an opportunity for people and planet? An interactive, student-led project focusing on prevention and agency in physician’s climate communication
Source: GMS J Med Educ. 2023 May 15;40(3):Doc34. doi: 10.3205/zma001616 (PMC10291343; doi:10.3205/zma001616)
Supplement: Initial concept paper of the elective “Climate-sensitive health counselling”, Giessen, for the winter term 2020/21 [translated from the original German version] [file JME-40-34-s-001.pdf]

Attachment 1: Initial concept paper of the elective “Climate-sensitive health counselling”, Giessen, for the winter term 2020/21 [translated from the original German version]

Attachment 1 to Fülbert H, Schäfer LN, Gerspacher LM, Bösner S, Schut C, Krolewski R, Knipper M. *Elective course “climate-sensitive health counselling”: Prevention as an opportunity for people and planet? An interactive, student-led project focusing on prevention and agency in physician’s climate communication.* GMS J Med Educ. 2023;40(3):Doc34. DOI: 10.3205/zma001616

Concept paper
Elective “Climate-sensitive health counselling”
Clinical elective at the Faculty 11 - Human Medicine at the Justus Liebig University Giessen

1. Why is an elective "Climate-sensitive consultation" relevant for the Faculty of Medicine at JLU Giessen?
2. Goals of the new elective Climate-sensitive consultation.
3. Implementation and procedure
4. Expectations and outlook
5. References

1. Why is an elective "Climate-sensitive consultation" relevant for the faculty of medicine at JLU Giessen?

Climate change is the greatest challenge of our time as it affects all areas of human life on our planet Earth. However, in medicine, and here we should mention teaching in particular, its effects on the human body are still little discussed. We believe that science-based and interdisciplinary teaching of the effects of climate change on the body and health is of enormous importance in the study of medicine. This is because climate change impacts disease patterns in every discipline. As future physicians, we therefore see an urgent opportunity to influence the lifestyles of our patients in a climate-friendly way through adapted prevention work. The connection between climate-damaging behavior and modern civilization diseases such as obesity, type 2 diabetes, cardiovascular diseases, and others has been established many times and discussed in the scientific debate. Therefore, we perceive it as essential to modernize teaching and to join the consensus: Health protection means climate protection[1] . This refers especially to a permanent integration of climate change into the teaching content of the clinical study section.
Secondly, it cannot be denied that climatic conditions have already changed in this country.[2] Thus, a change in infrastructure within the health care delivery system must also be addressed.
Students will have the opportunity to learn about current research on this topic, to engage with it, and, encouraged by this impetus, to practically integrate their knowledge growth into everyday clinical practice.

2. Goals of the new elective Climate-sensitive consultation.

The aim of the elective is to provide students with competencies on both the content and the communicative-creative level on the topic of climate change and health. The focus should be especially on health prevention and science communication of the topic. As described above,

---

[1] 5th IPCC Report of 2014: Commision 2, Chapter 11 Human Health: Impacts, Adaptation, and Co-Benefits see also: https://www.ipcc.ch/report/ar5/wg2/

[2] [2] Augustin, J., Sauerborn, R. et al. (2016): Gesundheit. In: Guy Brasseur et al.: *Klimawandel in Deutschland*. Springer, Berlin Heidelberg, ISBN 978-3-662-50397-3

we as future physicians see ourselves as important actors in communicating and mitigating the climate crisis.[3]

In the long term, the elective should be the prerequisite to enable students to work in a "Climate Service", e.g. in the university hospital. We see the task of the "Climate Service" in giving interested patients the opportunity to learn more about their illness in connection with climate change and to show them options how they can counteract climate change and their illness.

In addition, we would like to develop a concept together with doctor's offices in Giessen (especially family doctors and pediatricians) to offer climate-sensitive health counselling also in the outpatient context and possibly integrate it into the regular patient consultation, as already offered in some places[4] .

3. Implementation/process

The elective is scheduled to meet in 8-seminar sessions during the semester for 1.5 hours each week on Mondays from 6:15 - 7:45 pm. During the first 45 minutes, speakers will present a special topic. At this point, it is especially important to us to convey how climate change is related to cardiovascular disease, respiratory disease, nutrition and the immune system. The focus should always be on prevention work and appropriate communication of these topics. In the search for speakers, we want to focus primarily on local structures and, as far as possible, cooperate with the individual disciplines and institutes of Department 11 and, in general, with the University of Giessen. It would make sense to cooperate in the sense of interdisciplinarity for example with the faculties of nutritional sciences and psychology/medical psychology. In the second section, the students will compile and reflect on the information presented. Here, they will focus on topics that are particularly important for them as future physicians in the context of a patient interview. In teams of two, minutes are taken during the discussion, with two to three guiding questions being identified by the student team. The creation of a short, written portfolio will be assessed and graded as evidence of achievement.

The elective closes with a concluding session during which we will practice patient interviews using case studies from a family doctor's medical practice.

4. Expectations and outlook

In order to check the implementation of the new elective and to receive feedback, an evaluation by means of questionnaires will be carried out.

As can also be seen in the engagement of medical students with groups such as Health for Future, Doctors for Future, or participation in the Planetary Health Academy, there is a need to address the intersection of climate change and health in academic teaching as well as to provide a platform for discourse. We strongly believe that the elective will be met with great interest.

Prof. Dr. med. Michael Knipper will supervise the elective as the person responsible for teaching. The student contact person is Laura Gerspacher.

---

[3] [3] Robert-Koch-Institut (2013): Klimawandel und Gesundheit: Allgemeiner Rahmen zu Handlungsempfehlungen für Behörden und weitere Akteure in Deutschland (PDF).

[4] **Dr. Ralph Krolewski** (family doctor in Gummersbach, Bavaria) already offers a so-called "climate consultation". His work serves as a model for us; he is requested by us as a speaker.

Key points summarized

- Elective “Climate-sensitive health counselling” - the intersection of climate change and health.
- 8 weekly seminar units, Mondays, 18:15 - 19:45 (1,5 SWS) starting on 19.04.2021-21.06.21, plus one final concluding session on Saturday the 26.06.2021 (extent: 5 hours), maximum 25 participants
- Performance record: Creation of a portfolio of one seminar session

5. Literature

1) 5th Intergovernmental Panel on Climate Change (IPCC) Report of 2014: Commision 2, Chapter 11 Human Health: Impacts, Adaptation, and Co-Benefits see also: https://www.ipcc.ch/report/ar5/wg2/

2) Augustin, J., Sauerborn, R. et al. (2016): Gesundheit. In: Guy Brasseur et al.: Klimawandel in Deutschland. Springer, Berlin Heidelberg, ISBN 978-3-662-50397-3

3) Robert-Koch-Institut (2013): Klimawandel und Gesundheit: Allgemeiner Rahmen zu Handlungsempfehlungen für Behörden und weitere Akteure in Deutschland (PDF)
sowie
Bundesgesundheitsblatt, Ausgabe 5/2019: Gesundheitliche Herausforderungen des Klimawandels

4) Dr. Ralph Krolewski (Hausarzt in Gummersbach, NRW) bietet eine sogenannte “Klimasprechstunde” schon an. Seine Arbeit dient uns als Vorbild; er wird von uns als Referent angefragt.
