# Supplementary material for: Elective course “Climate-sensitive health counselling” – prevention as an opportunity for people and planet? An interactive, student-led project focusing on prevention and agency in physician’s climate communication
Source: GMS J Med Educ. 2023 May 15;40(3):Doc34. doi: 10.3205/zma001616 (PMC10291343; doi:10.3205/zma001616)
Supplement: Concept and performance record of the elective “Climate-sensitive health counselling” Giessen; winter term 2021/22 [translated from the original German version] [file JME-40-34-s-002.pdf]

Attachment 2: Concept and performance record of the elective “Climate-sensitive health counselling” Giessen; winter term 2021/22 [translated from the original German version]

Attachment 1 to Fülbert H, Schäfer LN, Gerspacher LM, Bösner S, Schut C, Krolewski R, Knipper M. *Elective course “climate-sensitive health counselling”: Prevention as an opportunity for people and planet? An interactive, student-led project focusing on prevention and agency in physician’s climate communication.* GMS J Med Educ. 2023;40(3):Doc34. DOI: 10.3205/zma001616

Elective *“Climate-sensitive Health Counselling”*:

The intersection of climate change and health

In the winter semester 2021/22 at Justus Liebig University Giessen

JUSTUS-LIEBIG-UNIVERSITÄT GIESSEN

SPC Schwerpunktcurriculum Global Health

HEALTH FOR FUTURE

Procedure:
Mondays from 6:15-7:45 p.m., weekly **beginning Nov. 1, 2021** plus one **concluding session on Jan. 29, 2022** (9 a.m.-3 p.m.)

Content:
The climate crisis threatens our health. This connection is undisputed in science. You haven't heard about this yet, even though you are studying medicine? As future physicians, we are aware of our position as important mediating and mitigating actors regarding the climate crisis and would like to educate ourselves in this regard.
The aim of the elective is to provide students with competencies on both the content and the communicative-creative level on the topic of climate change and health. The focus should be particularly on health prevention and science communication of the topic.

In terms of content, we will address the following in the elective:

⇒ Fundamentals of climate change
⇒ Nutrition
⇒ Heat, air pollution
⇒ Lung Health
⇒ Gynecology
⇒ Infectiology
⇒ Mental health
⇒ Public Health
⇒ The role of the health sector
⇒ Climate communication/behavior change

Proof of Performance:
Portfolio for one elective session

Registration:
Send to giessen@healthforfuture.de; *subject*: Klimasprechstunde (= elective “Climate sensitive health counselling”); please indicate name and matriculation number.
