# Supplementary material for: Elective course “Climate-sensitive health counselling” – prevention as an opportunity for people and planet? An interactive, student-led project focusing on prevention and agency in physician’s climate communication
Source: GMS J Med Educ. 2023 May 15;40(3):Doc34. doi: 10.3205/zma001616 (PMC10291343; doi:10.3205/zma001616)
Supplement: Schedule of the elective “Climate-sensitive health counselling” Giessen, winter semester 2021/22 [translated from the original German version] [file JME-40-34-s-003.pdf]

Attachment 3: Schedule of the elective “Climate-sensitive health counselling” Giessen, winter semester 2021/22 [translated from the original German version]

Attachment 3 to Fülbert H, Schäfer LN, Gerspacher LM, Bösner S, Schut C, Krolewski R, Knipper M. *Elective course “climate-sensitive health counselling”: Prevention as an opportunity for people and planet? An interactive, student-led project focusing on prevention and agency in physician’s climate communication.* GMS J Med Educ. 2023;40(3):Doc34. DOI: 10.3205/zma001616

Elective "Climate-sensitive Health Counselling":
The intersection of climate change and health
Winter semester 2021/22, Justus Liebig University Giessen

JUSTUS-LIEBIG-UNIVERSITÄT GIESSEN

SPC Schwerpunktcurriculum Global Health

HEALTH FOR FUTURE

Schedule (as of 29.10.2021)

Mondays from 6:15 p.m. - 7:45 p.m., plus a final concluding session on Jan. 29, 2022 from 9 a.m. - 3 p.m.

01.11.2021: Introduction

08.11.2021: Lung health and allergies in the context of climate change

11/15/2021: The new cardiovascular risk factors - noise and particulate matter as triggers of cardiovascular disease.

11/22/2021: Opportunities of a plant-based diet for individual as well as planetary health.

11/29/2021: The particular vulnerability of children in the context of the climate crisis.

12/06/2021: Trauma sequelae and depression - An impact of the climate crisis?

13.12.2021: The risk factor heat

10.01.2022: Transformative action on the individual and socio-political level

17.01.2022: Anesthesia and environmental protection in hospitals - example from the university hospital of Gießen and Marburg (UKGM)

Concluding session on Saturday, Jan. 29, 2022, from 9 a.m. - 3 p.m:
Climate-sensitive health counselling in practice and behavioral psychology.
