# Supplementary material for: Elective course “Climate-sensitive health counselling” – prevention as an opportunity for people and planet? An interactive, student-led project focusing on prevention and agency in physician’s climate communication
Source: GMS J Med Educ. 2023 May 15;40(3):Doc34. doi: 10.3205/zma001616 (PMC10291343; doi:10.3205/zma001616)
Supplement: Expanded concept of the elective “Climate-sensitive health counselling”, Marburg, winter semester 2021/22 [translated from the original German version] [file JME-40-34-s-004.pdf]

# Attachment 4: Expanded concept of the elective "Climate-sensitive health counselling", Marburg, winter semester 2021/22 [translated from the original German version]

Attachment 4 to Fülbert H, Schäfer LN, Gerspacher LM, Bösner S, Schut C, Krolewski R, Knipper M. *Elective course "climate-sensitive health counselling": Prevention as an opportunity for people and planet? An interactive, student-led project focusing on prevention and agency in physician's climate communication.* GMS J Med Educ. 2023;40(3):Doc34. DOI: 10.3205/zma001616

# Elective for students in the clinical section of their studies: "Climate change and health"

In the winter semester 2021/22 at the University of Marburg – Version: 07.11.2021

## Content


1. general information....................................................................................................1
2. organizational and contact details...............................................................................2
3. procedure and dates.....................................................................................................3
4. exam performance information....................................................................................5
5. literature and further information...............................................................................6


## 1. General information

Climate change is characterized as the greatest threat to human health in the 21st century by the WHO (WHO 2021: 2). Droughts, heat waves and other extreme weather events will become more frequent, while rising temperatures and rising sea levels could make many populated areas uninhabitable and serve as infectious disease spreading factors (Werner et al. 2020: 2767f.).
According to a study by the European Environment Agency in September 2020, 400,000 people in the EU alone are expected to die prematurely each year as a result of air pollution (European Environment Agency 2020: n.d.). Worldwide, the WHO assumes 7 million deaths from air pollution (Kuehn 2014: 1486). Heat as a cause of illness and death is not included in either study. Nevertheless, the heat wave of 2003, which is now estimated to have caused more than 70,000 deaths in Europe, is an example of the impact that more frequent extreme weather events can have on health (Robine et al. 2008: 177).
In addition, it is assumed that the healthcare system is responsible for a not inconsiderable share of pollutant emissions in many countries. In Germany, this share is estimated at 5% (Lenzen et al. 2020: e275).
The elective with a total of 3 SWS [3 weekly hours/semester] is designed to provide students with an insight into 'Planetary Health', i.e. the connections between the topics of climate change and health. On the one hand, the health effects of the climate crisis are discussed, on the other hand, how the health sector exacerbates this crisis.

**Scope for recognition as an elective at the University of Marburg (3 SWS):**

1. Elective course Climate-sensitive Consultation at JLU Giessen (1.5 SWS)
   11/01/21 - 01/24/22, Mondays from 6:15 to 7:45 p.m. + block session on Saturday, 01/29.
2. Participation in the additional Marburg introductory and final sessions
   (08.11 [20:00-21:00] + 31.01 [16:15-17:45 or 18:15-19:45])
3. Self-paced viewing of additional videos from the Planetary Health Academy.
   The list is located later in the document.
4. Examination performance: Essay (see point 4)

**Explanation:**
The student-organized elective Climate-sensitive health counselling at the JLU Giessen (supervised by Prof. Dr. Michael Knipper) comprises a total of 9 evenings, during which the practical or micro level of the topic is examined. For this purpose, experts will be invited to each of the 9 evening meetings and present a topic. Afterwards, specific questions on the topic are worked on in small groups, which are then presented in the plenary. Towards the end of the semester, a final concluding session takes place to reflect on and apply what has been learned.
The Planetary Health Academy (PHA; https://planetary-health-academy.de) is organized by the German Alliance for Climate Change and Health (Deutsche Allianz Klimawandel und Gesundheit, KLUG) since the summer semester 2019. As the elective in this winter semester focuses primarily on clinical aspects, lectures from previous online events offered by the PHA are referred to for presenting and discussing further connections between climate change and health, especially on a macro level. A listing of the videos can be found on page 4.
Furthermore, there will be two additional meetings for the participants from Marburg: One at the beginning of the elective and another one at the end of the elective for reflection in smaller groups, clarification of open questions and further information on the examination performance.

## 2. registration, organizational matters and contact details

**Enrollment and Performance Records:**

➔ Registration via mail with the reference that you come from Marburg, matriculation number and semester to giessen@healthforfuture.de .
For students from Marburg 10 places are provided.

Send proof of performance by 3/31/22 to *[personal email addresses removed]*, who will grade the assignment.

**Access to the kmed folder:**
WF “Climate-sensitive health counselling” Giessen:
*Please check if the access is working!*
*Additional information for participants from Marburg will be available in a subfolder provided.*

**Contact details:**
Organizing team Marburg (Please always write to both addresses):
*[personal email addresses removed]*

Organizing team Giessen:
giessen@healthforfuture.de

## 3. procedure and dates

### Additional introduction date Marburg - mandatory - via BigBlueButton:

| | |
|---|---|
| **08.11.21**<br>**20:00 - 21:00** | Introduction and<br>Additional information<br>Marburg |

### Elective Climate Consultation Giessen - 2 missing dates -.
### WebEx link will be announced by mail

| | | |
|---|---|---|
| **01.11.21 18:15 - 19:45** | Introduction | Dr. Alina Herrmann, Heidelberg |
| **08.11.21 18:15 - 19:45** | Lung health | Dr. Christian Grah, Berlin |
| **15.11.21 18:15 - 19:45** | Cardiovascular diseases | Prof. Dr. Thomas Münzel, Mainz |
| **22.11.21 18:15 - 19:45** | Nutrition | Dr. Sebastian Göbel, Hermaringen |
| **29.11.21 18:15 - 19:45** | Pediatrics | Prof. Dr. Klaus-Peter Zimmer, Gießen |
| **06.12.21 18:15 - 19:45** | Mental illness | Prof. Dr. Johannes Kruse, Gießen |
| **13.12.21 18:15 - 19:45** | Heat | Prof. Dr. Claudia Traidl-Hofmann,<br>Augsburg / Munich- Schwabing |
| *20.12.21 - 03.01.22* | *Christmas vacations* | - |
| **10.01.22 18:15 - 19:45** | Transformative action | Dr. Martin Herrmann, KLUG |
| **17.01.22 18:15 - 19:45** | Anesthesia & Climate<br>Protection in<br>Hospitals | Dr. Ferdinand Lehmann, Giessen &<br>Mattis Keil, Bremen |
| **24.01.22 18:15-19:45** | *Alternate date* | *Expected free* |
| **29.01.21 09:00-15:00**<br>Saturday Compulsory<br>attendance! | Climate-sensitive<br>consultation in practice,<br>change narrative &<br>behavioral psychology. | Dr. Christina Schut, Giessen &<br>Dr. Ralph Krolewski, Gummersbach |

### Additional concluding session, Marburg (compulsory) via BigBlueButton

| | |
|---|---|
| **31.01.22 16:15 - 17:45** | concluding session 1 *OR* |
| **31.01.22 18:15 - 19:45** | concluding session 2 |

## Planetary Health Academy Lecture Videos - Viewing Mandatory

| No. | Title | Duration | Link |
|---|---|---|---|
| 1 | 10 years to transform the future of humanity - or destabilize the planet | 0:07:46 | https://www.youtube.com/watch?v=8Sl28fkrozE |
| 2 | Planetary Health - a New Scientific Discipline (PHA 3-2) | 1:29:00 | https://www.youtube.com/watch?v=VDNrORHd39A |
| 3 | Transdisciplinary Perspectives (PHA 1-5). | 1:41:01 | https://www.youtube.com/watch?v=cKG7KdbAE9k |
| 4 | Social Tipping Elements | 0:34:47 | https://www.youtube.com/watch?v=cRwClDeM-18 |
| 5 | Gender and Global South Perspective (PHA 2-4) | 1:34:27 | https://www.youtube.com/watch?v=_s79sO0V_yk |
| 6 | Urban Development and Planetary Health (PHA 2-5). | 1:31:05 | https://www.youtube.com/watch?v=o4S5WgxtoA4 |
| 7 | Communication (PHA 1-6) | 1:45:52 | https://www.youtube.com/watch?v=Zjjp-AsD9ok |
| 8 | Examples of Transformation (PHA 2-6) | 1:58:17 | https://www.youtube.com/watch?v=glP6K2M4s3k |

**Voluntary appointments for exchange (VLs, questions, ...) - optional**
**Presence / Online: depending on interest**

| | |
|---|---|
| **12.01.22 18:15** Wednesday | *optional* extra session |
| **24.01.22 18:15** Monday | *optional* extra session |

**OPTIONAL: Lecture Series Climate Crisis and Health Marburg - Partial overlap with the elective**

https://www.go-unimarburg.org/ringvorlesung-klimakrise-und-gesundheit/

## 5. information about the examination performance

The examination performance represents a 3–5-page essay (number of pages without cover sheet, table of contents, bibliography, affidavit) on a topic either chosen by the student or selected from the attached list. The topic is to be addressed using a self-selected research question. The evaluation will be done by Prof. Dr. Bösner from the Institute of General Medicine. The submission date can be found under point 2.

Font to be selected is Arial or Times New Roman, font size 12, justified, line spacing 1.5 (for quotes >4 lines indented, font size 11, line spacing 1.15). Page spacing is 2.5 cm at top, 2 cm at bottom, 2 cm at left, 2 cm at right. Page numbers are to be inserted.

The following information must be provided on the cover sheet: University, department, name of the course, own name, matriculation number, address, e-mail address (@students), semester, lecturer, semester of the examination, title (+subtitle) of the essay, place and date.

The citation method can be chosen freely but should be based on the common formats. Direct and indirect quotations must be uniformly identified, and the literature used must be uniformly and comprehensibly indicated in the bibliography. All in all, an essay represents a freer form of personal examination of a topic, so that one's own points of view and opinions can certainly be included.

Suggested topics should be discussed with Prof. Bösner in advance.

**Suggested topics for the term paper**

- The 2003 heat wave in Europe and its consequences
- Global inequalities, climate justice and health
- Heat protection systems
- Heat and Urban Development
- Climate-neutral healthcare sector
- Gender and Planetary Health
- Climate protection in the hospital / the practice / the nursing home
- Impact of the climate crisis on a specific organ system (including issues that are not part of the elective, e.g. dermatology, trauma surgery, infectious diseases, neurology, etc.)
- "Climate-sensitive health counselling"
- Medical ethics and the climate crisis
- Suggesting your own topics is encouraged!

**References:**

European Environment Agency (08.09.2020): Tackling pollution and climate change in Europe will improve health and well-being, especially for the most vulnerable. Online verfügbar unter / Available from: https://www.eea.europa.eu/highlights/tackling-pollution-and-climate-change, zuletzt geprüft am 13.09.2020.

Kuehn, Bridget M. (2014): WHO: More than 7 million air pollution deaths each year. In: *JAMA* 311 (15), S. 1486. DOI: 10.1001/jama.2014.4031 .

Lenzen, Manfred; Malik, Arunima; Li, Mengyu; Fry, Jacob; Weisz, Helga; Pichler, Peter-Paul et al. (2020): The environmental footprint of health care: a global assessment. In: *The Lancet Planetary Health* 4 (7), e271-e279. DOI: 10.1016/S2542-5196(20)30121-2 .

Robine, Jean-Marie; Cheung, Siu Lan K.; Le Roy, Sophie; van Oyen, Herman; Griffiths, Clare; Michel, Jean-Pierre; Herrmann, François Richard (2008): Death toll exceeded 70,000 in Europe during the summer of 2003. In: *Comptes rendus biologies* 331 (2), S. 171–178. DOI: 10.1016/j.crvi.2007.12.001 .

Werner, Doreen; Kowalczyk, Stefan; Kampen, Helge (2020): Nine years of mosquito monitoring in Germany, 2011-2019. with an updated inventory of German culicid species. In: *Parasitology research* 119 (9), S. 2765–2774. DOI: 10.1007/s00436-020-06775-4 .

World Health Organisation (Hg.) (2021): COP26 Special Report on Climate Change and Health. The Health Argument for Climate Action. Genf: WHO.

**General info on climate change and health:**

The Lancet Countdown on Climate Change and Health:
https://www.thelancet.com/countdown-health-climate

Facts about climate change and health at Health for Future:
https://healthforfuture.de/klima-und-gesundheit/

German Climate Change and Health Alliance (KLUG e.V.):
https://www.klimawandel-gesundheit.de/

WHO:
https://www.who.int/health-topics/climate-change

**Recommended reading (not a must, have 2 copies privately to borrow temporarily):**

Traidl-Hoffmann, Claudia; Schulz, Christian; Herrmann, Martin; Simon, Babette (Hg.) (2021): Planetary Health. Klima, Umwelt und Gesundheit im Anthropozän. Berlin: MWV. ISBN: 978-3-95466-650-8.
