# Supplementary material for: Elective course “Climate-sensitive health counselling” – prevention as an opportunity for people and planet? An interactive, student-led project focusing on prevention and agency in physician’s climate communication
Source: GMS J Med Educ. 2023 May 15;40(3):Doc34. doi: 10.3205/zma001616 (PMC10291343; doi:10.3205/zma001616)
Supplement: Exemplary survey instrument for the evaluation of the elective “Climate-sensitive health counselling”, summer semester 21 [file JME-40-34-s-005.pdf]

Attachment 5: Exemplary survey instrument for the evaluation of the elective "Climate-sensitive health counselling", summer semester 21

Attachment 5 to Fülbert H, Schäfer LN, Gerspacher LM, Bösner S, Schut C, Krolewski R, Knipper M. *Elective course "climate-sensitive health counselling": Prevention as an opportunity for people and planet? An interactive, student-led project focusing on prevention and agency in physician's climate communication.* GMS J Med Educ. 2023;40(3):Doc34. DOI: 10.3205/zma001616

Evaluation of the elective course “climate-sensitive health counselling” in the summer semester 2021.

Surveyed twice: after half of the sessions and at the end of the course; data on the overall evaluation derived from the survey at the end of the course.

Part 1 - For each individual Session:
Session [nr]. Date: [title] I rate as:
very good (5) - good (4) - alright (3) - bad (2) - very bad (1) - no indication

This is what I would like to share with you you regarding the session: [Free text]

Part 2 – Final concluding session:
I rate the whole concept of the concluding session as:
very good (5) - good (4) - alright (3) - bad (2) - very bad (1) - no indication

I rate the part "Behavioral psychology and motivational interviewing" as:
very good (5) - good (4) - alright (3) - bad (2) - very bad (1) - no indication

I rate the part "Integration of climate change into everyday practice“ as:
very good (5) - good (4) - okay (3) - bad (2) - very bad (1) - no indication

This is what I would like to share with you you regarding the session / This is what I liked best or least: [free text]

Part 3 - Overall evaluation of the elective:
I rate the entire elective as:
very good (5) - good (4) - okay (3) - poor (2) - very poor (1) – no indication

I rate the structure of the whole elective as:
very good (5) - good (4) - alright (3) - bad (2) - very bad (1) - no indication

I evaluate the organization and the course of the whole elective as:
very good (5) - good (4) - alright (3) - bad (2) - very bad (1) - no indication

I rate my increase in knowledge as:
very high (5) - high (4) - somewhat (3) - low (2) - none (1) – no indication

The elective motivates me to continue studying climate change and health:
very (5) - good (4) - somewhat (3) - a little (2) - not at all (1) - no indication

I particularly liked: [Free text]

I didn't think that was so good / fell short: [Free text]

This is what else I would like to share with you: [Free text]
